# Photonuclear Population of ^229m,g^Th in Th‐Doped Crystals Toward Nuclear Clock Development

**DOI:** 10.1002/advs.202523384

**Published:** 2026-02-27

**Authors:** Hao‐Yang Lan, Di Wu, Mei‐Zhi Wang, Zhong‐Yi Chen, Jian‐Feng Lv, Yu‐Hui Xia, Zhe‐Nan Wang, Wen Luo, Xin‐Lu Xu, Xue‐Qing Yan

**Affiliations:** ^1^ State Key Laboratory of Nuclear Physics and Technology and CAPT Peking University Beijing China; ^2^ Beijing Laser Acceleration Innovation Center Beijing China; ^3^ School of Nuclear Science and Technology University of South China Hengyang China

**Keywords:** nuclear clock, nuclear excitation, photonuclear reactions

## Abstract

Efficient population of the 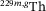 is essential for advancing nuclear clock technology, whose progress faces strategic challenges related to the finite legacy supply and competing high‐value medical applications of 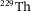. This study proposes a novel approach to populate 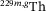 via photonuclear reactions in thorium‐doped crystals. Theoretical calculations were performed to evaluate the photonuclear reaction cross‐sections of four thorium isotopes 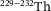, which show 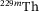
‐related production cross‐sections reaching hundreds of millibarns. Monte Carlo simulations further analyzed the time‐wavelength distributions of 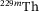 radiative decay signals and Cherenkov radiation backgrounds in thorium‐doped ${\mathrm{CaF}}_{2}$, ${\mathrm{SrF}}_{2}$, and LiF crystals under bremsstrahlung irradiation, revealing the correlation between the signal‐to‐noise ratio (SNR) and incident electron parameter. The results show that ${\mathrm{SrF}}_{2}$ crystals achieved an SNR of 10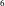 under irradiation with bremsstrahlung driven by 1 C electrons at ∼40 MeV, outperforming other materials. Compared to VUV laser direct excitation and X‐ray resonant scattering pumping, this method exhibits unique advantages, including target material flexibility, broad light source compatibility, and higher excitation rates. Moreover, by enabling the conversion of abundant 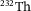 and 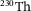 into 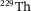
‐doped crystals, photonuclear reactions offer a decentralized production route that is independent of the finite 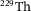 supply derived from regulated 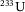 inventories. These findings indicate that the photonuclear population is a promising pathway for efficient 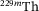 production and characterization, effectively mitigating future supply constraints for fundamental research.

## Graphical abstract

This study demonstrates a novel photonuclear approach for producing the nuclear clock isomer ^229m^Th in thorium‐doped crystals, overcoming current supply limitations. Through cross‐section calculations and Monte Carlo simulations, we identify optimal crystal materials and irradiation conditions, achieving high signal‐to‐noise ratios. This method offers a flexible, decentralized production route independent of regulated 233U inventories, advancing nuclear clock technology and fundamental research.

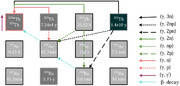

## 1 Introduction

The nuclear isomer 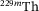 is well‐known for its exceptionally low excitation energy of ∼8 eV, which is comparable to the energy of the atomic transitions used in atomic clocks [1, 2, 3, 4, 5]. Since ultraviolet light sources can reach such energy, 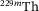 has long been expected to be used to develop an advanced nuclear clock. This nuclear clock is predicted to have an uncertainty of approximately 10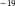, comparable to the most accurate optical atomic clocks. Unlike conventional optical atomic clocks, which rely on fragile electronic transitions, a 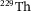
‐based nuclear clock leverages robust nuclear energy levels, enabling unprecedented resilience to environmental perturbations [6]. This resilience promises breakthroughs in geodesy, seismology, and fundamental physics—from probing variations in fundamental constants to detecting dark matter [7, 8, 9, 10, 11, 12, 13, 14]. In particular, a solid‐state nuclear clock [12, 15] offers the advantage of simultaneously interrogating a large ensemble of 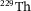 nuclei embedded in a vacuum ultraviolet (VUV) transparent crystal, enabling high signal strength, continuous operation, and potentially ultra‐high‐frequency stability without the complexity of ion trapping. However, realizing this potential hinges on two key challenges: efficiently populating the elusive 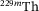 isomer, whose weak and uncertain transition hampers detection, and fabricating effective 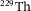
‐doped materials.

Over the past 50 years, researchers have explored various methods to achieve efficient excitation of 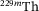 and its direct photon decay detection in multiple materials. The earliest method exploited α decay of the scarce 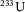 (half‐life $∼10^{5}$ years), but its low decay branching ratio to 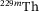 and the complex background noise proved difficult in direct photon decay detection [16, 17, 18, 19, 20, 21, 22]. Alternative approaches used proton spallation of Th/U targets to produce short‐lived parent nuclei of 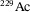 [23, 24] (half‐life 62 min) or 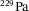 [25] and implant them into ${\mathrm{CaF}}_{2}$ or ${\mathrm{MgF}}_{2}$ crystals, yielding the first direct photon decay detection [26]. While spallation facilitates the 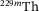 population and detection, it requires a large‐scale high‐energy (>100 MeV) proton accelerator and complex isotope isolation facilities. In recent years, advances in laser‐plasma acceleration technology have inspired researchers to propose the idea of using the collision of hot electrons with 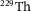 ions during laser‐target interactions to produce 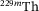 [27, 28, 29, 30], though unverified experimentally and difficult to apply in 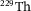
‐doped materials. Moreover, narrow‐band X‐ray bombardment of a 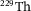 target can excite it to higher energy states above the isomer, rapidly transitioning to the metastable 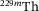. Experiments conducted at the Spring‐8 synchrotron radiation facility successfully pumps 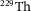 from the ground state to the second excited state at 29 keV [31, 32], achieving an efficient 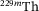 production rate of 2.5$\times 10^{4}$ $s^{-1}$ inside 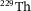
‐doped ${\mathrm{CaF}}_{2}$ crystals. Recently, advancements in the direct population of 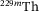 [33, 34], facilitated by state‐of‐the‐art VUV laser systems [35, 36] and innovative 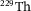 doping techniques [37, 38, 39], have significantly advanced the field, inspiring critical studies such as high‐precision spectroscopy referenced to the 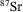 clock [40] and investigations into the “quenching effect” [32]. These efforts achieved an instantaneous excitation rate of $∼2\times 10^{7}$ $s^{-1}$. Although significant progresses have been made in the excitation of 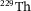, its further development in nuclear clock applications remains constrained by three major bottlenecks: shortages in raw 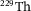 material supply, risks associated with doping radioactivity, and the limited operating power of VUV lasers. Currently, the global 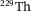 inventory remains severely limited, further strained by competing demands in nuclear medicine and strict control regulations of such special nuclear materials [41, 42, 43, 44, 45]. The primary application of 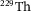 extracted from legacy inventories of 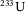 (whose use is strictly regulated due to its potential application in nuclear weapons) is the production of 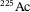 for targeted alpha cancer therapy. Even under optimistic assumptions, the annual yield of 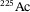 from this finite 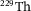 source (a total of 40 g within 3 years if fully extracted) is limited to roughly 2–3 Ci, which is orders of magnitude below projected global clinical demand. Consequently, 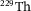 is a high‐value resource likely to be prioritized for medical production, which may constrain its accessible supply for fundamental research in the future. To address these challenges, advanced materials like 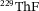 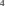 thin films [46] are being developed to expand the applications of nuclear clocks. Despite these innovations, the scarcity of 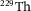 poses significant constraints on essential testing, such as studying the excitation and decay behavior of 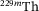 in diverse host materials and under environmental fluctuations [32, 47, 48, 49, 50, 51, 52]. Additionally, the limited operating power for VUV lasers is primarily a consequence of insufficient power spectral density. Current pulsed VUV sources face a fundamental trade‐off: systems like nanosecond four‐wave mixing [35, 36] offer high peak power but are plagued by GHz‐scale linewidths, which drastically dilute their spectral brilliance. Conversely, high‐harmonic generation frequency combs [40] achieve narrow linewidths but distribute their total power across millions of comb teeth, resulting in only 1 nW per tooth. In both cases, the power within the narrow bandwidth required for coherent nuclear excitation is prohibitively low. Consequently, developing an efficient 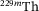 population method with target and beam flexibility is beneficial for the further proliferation of nuclear clock applications.

In this work, we propose to efficiently populate 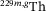 in crystals through photonuclear reactions around the giant dipole resonance (GDR) region. This approach is centered on a practical production pathway: converting readily available, high‐quality crystals doped with abundant thorium isotopes (e.g., 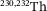) into active clock media containing 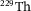. It thereby decouples early‐stage material development and testing from the constrained supply chain for purified 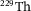. Our focus on populating the isomeric state 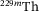 serves two critical purposes within this framework: (1) enabling rapid in situ study of the isomer's properties (e.g., lifetime, quenching) within candidate host crystals—an essential step for materials optimization, and (2) providing a direct spectroscopic fingerprint to verify and benchmark successful 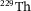 production. A systematic theoretical framework is established to analyze photonuclear pathways for 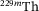 production and associated radioactive byproducts. Theoretical calculations of photonuclear reaction cross‐sections were performed for four thorium isotopes (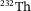, 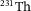, 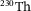, 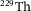), incorporating state‐of‐the‐art nuclear models. These cross‐section data were subsequently integrated into Monte Carlo simulations to quantify the yield of 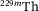
‐related radioactive residuals in thorium targets under bremsstrahlung irradiation from electron accelerators. For VUV detection schemes utilizing crystal‐hosted thorium isotopes, we quantified Cherenkov backgrounds from β‐emitting byproducts in thorium‐doped ${\mathrm{CaF}}_{2}$, ${\mathrm{SrF}}_{2}$, and LiF crystals. The signal‐to‐noise ratio (SNR) under different beam‐target configurations is investigated. It is shown that the SNRs for thorium‐doped ${\mathrm{SrF}}_{2}$ are better than the other cases, yielding an SNR of 10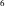 with irradiation of 1 C electrons of ∼40 MeV. Moreover, the prospect to convert abundant 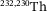 into 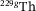 inside VUV transparent crystals is foreseen. This method offers distinct advantages, including flexibility in target fabrication and beam parameters, compatibility with broad‐spectrum bremsstrahlung sources, and high production rates, presenting a promising and complementary route for fabricating and characterizing 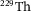
‐doped crystalline materials.

## 2 Results

### 2.1 Photonuclear Reaction Cross‐Sections and Yields

**FIGURE 1:**
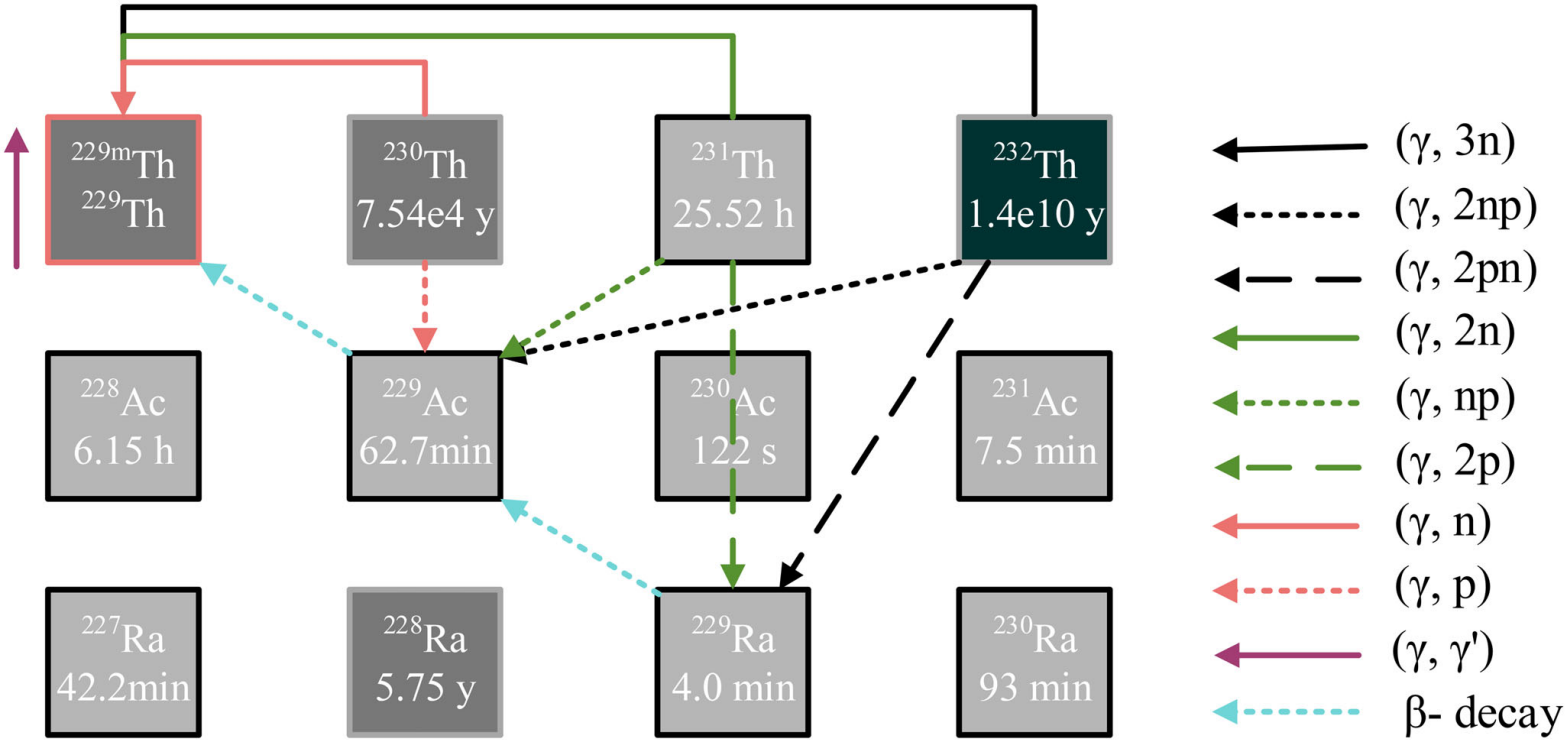
Schematic drawing of the 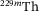 population by photonuclear reactions. The nuclear flow of photonuclear reactions on 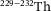 targets. The 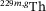 isotopes of interest can be produced directly from 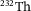
(γ, 3n), 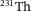
(γ, 2n), 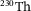
(γ, n), and 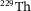
(γ, γ') reactions. The 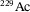 parent nucleus can be produced from 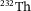
(γ, 2np), 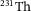
(γ, np), and 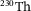
(γ, p) reactions. The 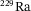 parent nucleus can be produced from 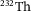
(γ, 2pn), and 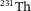
(γ, 2p) reactions.

The photonuclear reactions driven by MeV γ‐ray sources provide an effective pathway for populating the 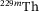 isomer. Above the particle separation threshold, the GDR—resulting from the out‐of‐phase oscillation between proton and neutron fluids—significantly enhances the probability of photon absorption and compound nucleus formation. The decay of the compound nucleus leads to the production of 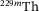 and its associated highly excited states through multiple photonuclear reaction channels, as illustrated in Figure 1. For instance, the 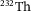
(γ, 3n) and 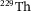
(γ, $\gamma^{\prime }$) reactions directly populate 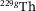 and 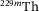, while the 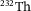
(γ, 2np) and 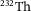
(γ, n2p) reactions produce 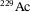 and 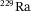, respectively. These isotopes subsequently decay into 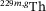 via $\beta^{-}$ decay chains. Theoretically, photonuclear reactions on thorium isotopes 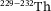 can generate all residuals of interest, including 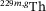, 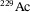, and 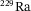.

**FIGURE 2:**
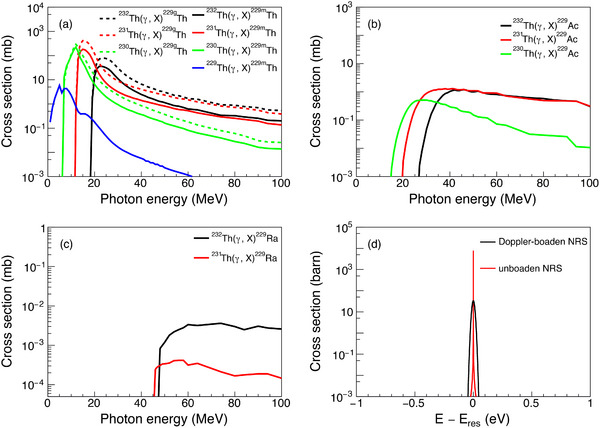
The cross‐sections of the photonuclear reactions that can produce 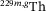 (a), 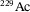 (b), and 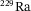 (c). (d) The NRS cross‐sections for the second excited state of 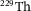 ($E_{res}$ = 29 keV) with and without consideration of Doppler broadening at room temperature.

To evaluate the reaction cross‐sections for the population of 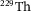
(including both its ground state, 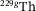, and its isomer, 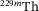), we performed calculations for photonuclear reactions on the four isotopes 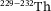, as described in Section 4.1. The calculated photonuclear reaction cross‐sections are presented in Figure 2a–c. The reaction threshold for direct 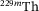 production decreases with increasing target atomic mass, with the 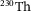
(γ, n)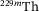 reaction reaching a peak cross‐section exceeding 100 mb. Similarly, the cross‐sections for producing the ground state 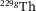 have been extracted and are shown for comparison, which are slightly larger than those for 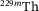. The thresholds for 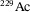 and 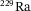 production are significantly higher, with cross‐sections typically below 1 mb and $10^{-2}$ mb, respectively. For comparison, Figure 2d shows the nuclear resonant scattering (NRS) cross‐sections. The unbroadened NRS cross‐section is calculated using the single‐level Breit‐Wigner formula as described in Ref. [31], while the Doppler‐broadened cross‐section is estimated by:

(1) $$\sigma_{NRS}\left(E\right)\approx {\left(\frac{\hbar c}{E_{res}}\right)}^{2}\frac{\pi^{3/2}}{\sqrt{2}\Delta }g\frac{\Gamma_{0}^{2}}{\Gamma }\exp\left(\frac{{\left(E-E_{res}\right)}^{2}}{2\Delta^{2}}\right)$$

where Γ is the width of the level at resonance energy $E_{res}$, $\Gamma_{0}$ is the partial width for transitions between $E_{res}$ and the ground state, ℏ is the Planck constant, c is the speed of light, $\Delta =E_{r}\sqrt{k_{B}T/Mc^{2}}$ is the Doppler width, $k_{B}$ is the Boltzmann constant, T is the absolute temperature, and M is the mass number of the nucleus.

Photonuclear population offers distinct advantages over the X‐ray NRS method. The photonuclear reaction's strength function around the GDR region spans from several MeV to tens of MeV, covering an energy range approximately ten orders of magnitude wider than the neV‐scale transitions accessible via NRS. This broad energy coverage enables the production of diverse nuclear states and activation products relating to 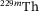 beyond the limitations of NRS. Furthermore, the technique eliminates the dependency on scarce 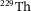 targets and expands material compatibility to a wider range of nuclei. Critically, it can utilize conventional γ‐ray sources, such as bremsstrahlung radiation from electron accelerators, bypassing the stringent requirements for ultra‐narrow‐bandwidth and high‐flux photon sources inherent to NRS. These attributes would collectively reduce experimental complexity and cost while enhancing applicability.

**FIGURE 3:**
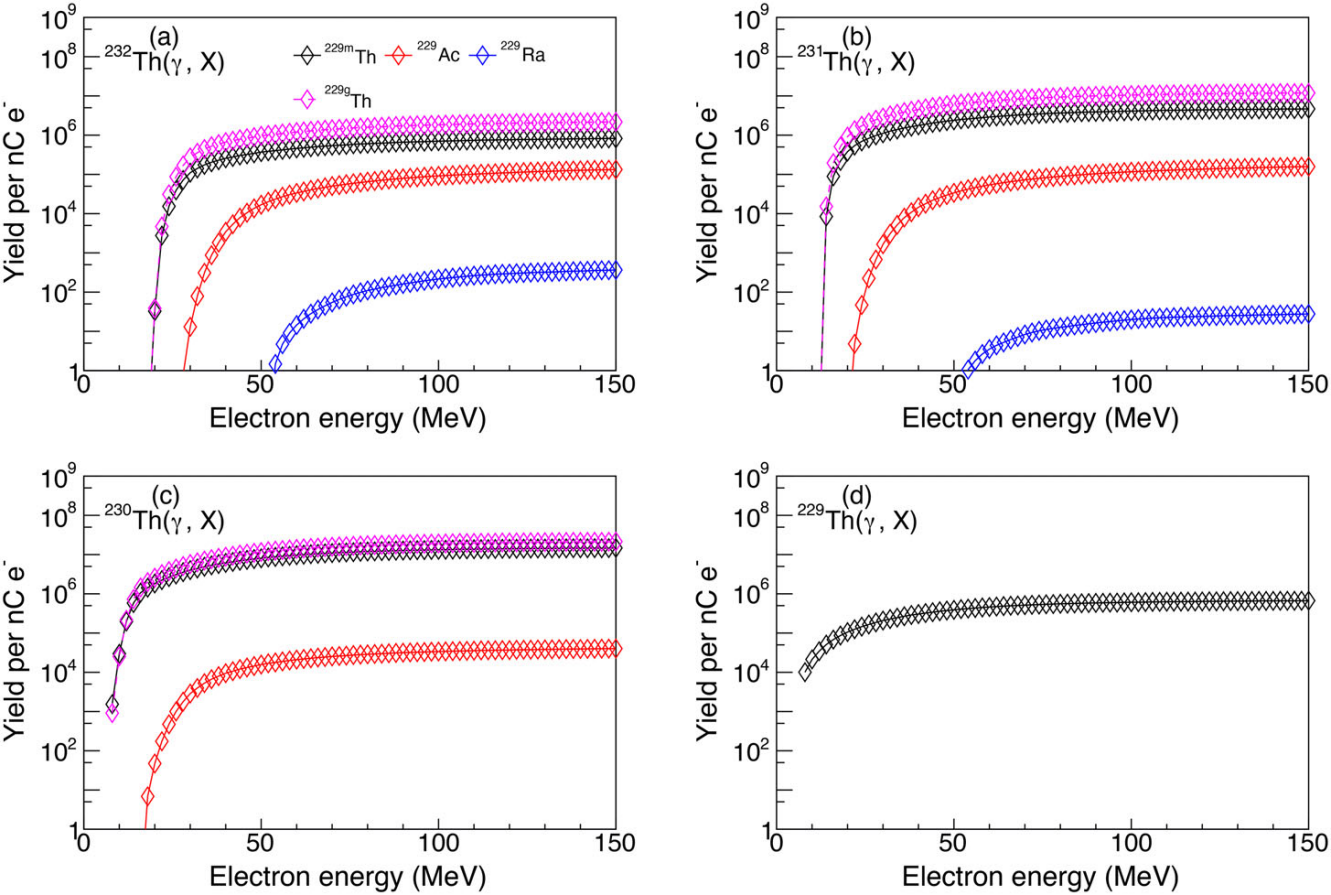
The 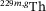, 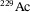, and 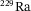 yields from the 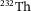 (a), 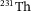 (b), 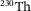 (c), and 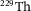 (d) targets under the irradiation of bremsstrahlung beam induced by electrons of different energies. The target thickness is 1 cm.

To evaluate the yields of residuals related to 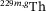 using the four isotopic thorium targets under electron‐driven bremsstrahlung irradiation, which is the most readily available γ ray source, Geant4 simulations were performed using the methods described in Section 4.2. The correlations between the yields of 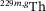, 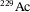, and 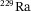 and the energy of incident electrons (with a charge of 1 nC) are presented in Figure 3. The yields of these isotopes increase with increasing electron energy, saturating above approximately 60 MeV. For a 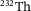 target, yields on the order of $10^{5}$, $10^{4}$, and $10^{2}$ are observed for 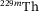, 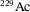, and 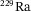, respectively. When using a 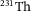 target, the yields are comparable to those of 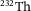, but saturation occurs at lower electron energies. For a 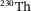 target, the yield of 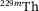 increases significantly, reaching approximately $10^{7}$, while the yield of 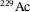 is lower than that observed for 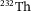 and 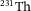. In contrast, for a 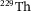 target, only the 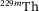 production channel remains active, yielding approximately $10^{5}$. Notably, the reaction cross‐sections and the corresponding simulated yields for total 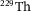 production are typically a factor of 1–2 higher than those for the isomer 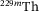 alone. Moreover, the yields also increase with greater target thickness, reaching saturation at a thickness of 4 cm. At this thickness, the maximum yields are increased by a factor of *1.2* compared to the case with a thickness of 1 cm.

### 2.2 Cherenkov Background and VUV Signal Emission in Doped Crystals

Although 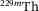 can be produced with high yield through photonuclear reactions, detecting its direct γ decay is difficult in a normal‐form target. This is mainly due to two reasons: First, the internal conversion ratio of 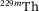 is as high as 10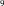, and the number of emitted photons is very few compared to the conversion electrons. Second, the energy of the emitted photons is as low as 8.3 eV, making it difficult to penetrate the thorium target before detection. To overcome such limitations, VUV crystals doped with thorium isotopes such as Th:${\mathrm{CaF}}_{2}$ were developed. When embedded in the crystal lattice, the internal conversion will be suppressed and 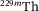 isomer will decay predominantly through purely γ‐ray emission with a half‐life of approximately 10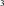 s. Moreover, the transmittance of VUV photons in such crystals is relatively high, which would not only ease the attenuation of 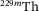 decay signals inside the target but also would facillitate a more efficient VUV laser excitation by minimizing the photon loss. Here, we further investigate the prospect to detect the direct photon signals by the photonuclear population of 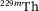 in three kinds of crystals (${\mathrm{CaF}}_{2}$, ${\mathrm{SrF}}_{2}$, and LiF) doped with the four above‐mentioned thorium isotopes, as described in Section 4.3.

**FIGURE 4:**
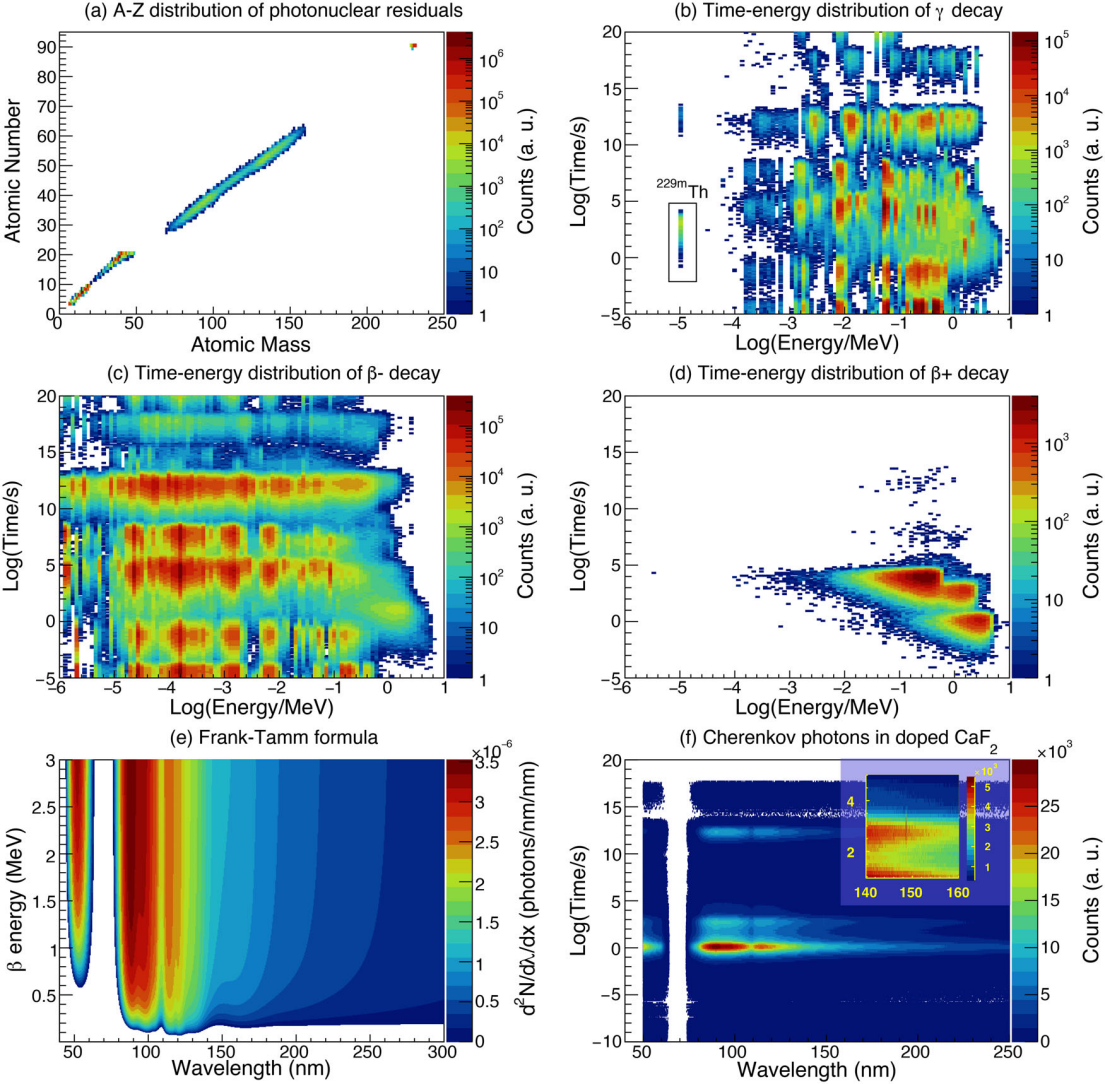
(a) The A‐Z distribution of photonuclear residuals produced in 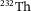:${\mathrm{CaF}}_{2}$ crystal at the electron energy of 60 MeV. (b) The time‐energy distribution of the decay γ rays of the photonuclear residuals. (c) The time‐energy distribution of the $\beta^{-}$ decays of the photonuclear residuals. (d) The time‐energy distribution of the $\beta^{+}$ decays of the photonuclear residuals. (e) The wavelength spectrum of emitted Cherenkov photons per track length inside a ${\mathrm{CaF}}_{2}$ crystal. (f) The wavelength‐time distribution of the emitted optical photons, involving 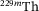 decay signal and Cherenkov backgrounds, for 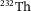:${\mathrm{CaF}}_{2}$ crystal at the electron energy of 60 MeV. The inset provides a magnified view of the region where the 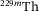 decay signal appears.

When a thorium‐doped crystal, such as the most representative and readily available 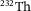:${\mathrm{CaF}}_{2}$, is irradiated by γ rays, photonuclear reaction residuals with atomic numbers ranging from 2 to 90 are produced, as shown in Figure 4a. The residuals resulting from the photonuclear reactions of Ca and F isotopes are concentrated around the atomic number of < 20, accounting for more than 90% of the total residual yield. In contrast, the residuals generated by the photofission of 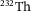 exhibit a double‐peak structure within the atomic number range of ∼25–65. Additionally, residuals from other photonuclear reactions of 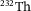, including 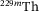, are clustered around an atomic number of 90. Most of these reaction residuals are radioactive and decay by emitting electrons, positrons, γ rays, or α particles. The energy‐time distribution of the emitted γ rays is illustrated in Figure 4b. Notably, the direct γ decay photons of 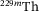, with an energy of 8.3 eV, are emitted with a half‐life of $10^{3}$ s in the crystalline environment.

When the charged particles from radioactive decay propagate through the crystal, optical photons ranging from extreme ultraviolet to visible wavelengths are generated via the Cherenkov effect. This effect constitutes a significant source of background in the detection of 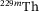 direct photon decay. For instance, in a ${\mathrm{CaF}}_{2}$ crystal (n=1.586), the threshold energies required to produce Cherenkov photons with λ=148 nm are approximately 0.15 MeV for electrons, 276 MeV for protons, and 1096 MeV for α particles. Since α particles from radioactive decays typically have energies below 10 MeV, the Cherenkov photons interfering with 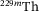 detection primarily originate from β decays. Figure 4c,d illustrate the energy‐time correlations of the electrons from $\beta^{-}$ decays and positrons from $\beta^{+}$ decays of radioactive residuals, respectively. A significant number of electrons and positrons with energies larger than 0.15 MeV are emitted from the radioactive decays in the time window of 10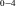 s, which will contribute to the Cherenkov background during the measurement of the direct photon decay signal of 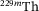.

The distribution of Cherenkov photons generated per unit track length dx by an energetic charged particle with a relativistic velocity β as a function of wavelength λ is often described using the Frank‐Tamm formula [53, 54]:

(2) $$\frac{d^{2}N}{dxd\lambda }=\frac{2\pi \alpha z^{2}}{\lambda^{2}}\left(1-\frac{1}{\beta^{2}n^{2}\left(\lambda \right)}\right)$$

where α is the fine structure constant, z is the charge of the propagating particle, and n(λ) is the refractive index at wavelength λ. Figure 4e shows the correlation of particle energy and λ against $d^{2}N/dxd\lambda$ for electrons traveling through a ${\mathrm{CaF}}_{2}$ crystal. The Cherenkov photons are most likely generated at λ<120 nm, and $d^{2}N/dxd\lambda$ is approximately $10^{-4}$ photons/${\mathrm{nm}}^{2}$ for the 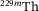 signal around λ∼148 nm. The wavelength and emission time distribution of Cherenkov photons resulting from the radioactive decay of photonuclear reaction residuals of 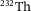:${\mathrm{CaF}}_{2}$ are shown in Figure 4f. The Cherenkov backgrounds emitted within the 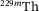 decay lifetime window of $10^{0-4}$ s and the wavelength of 148 nm are relatively low, allowing the observation of the direct decay photon.

**FIGURE 5:**
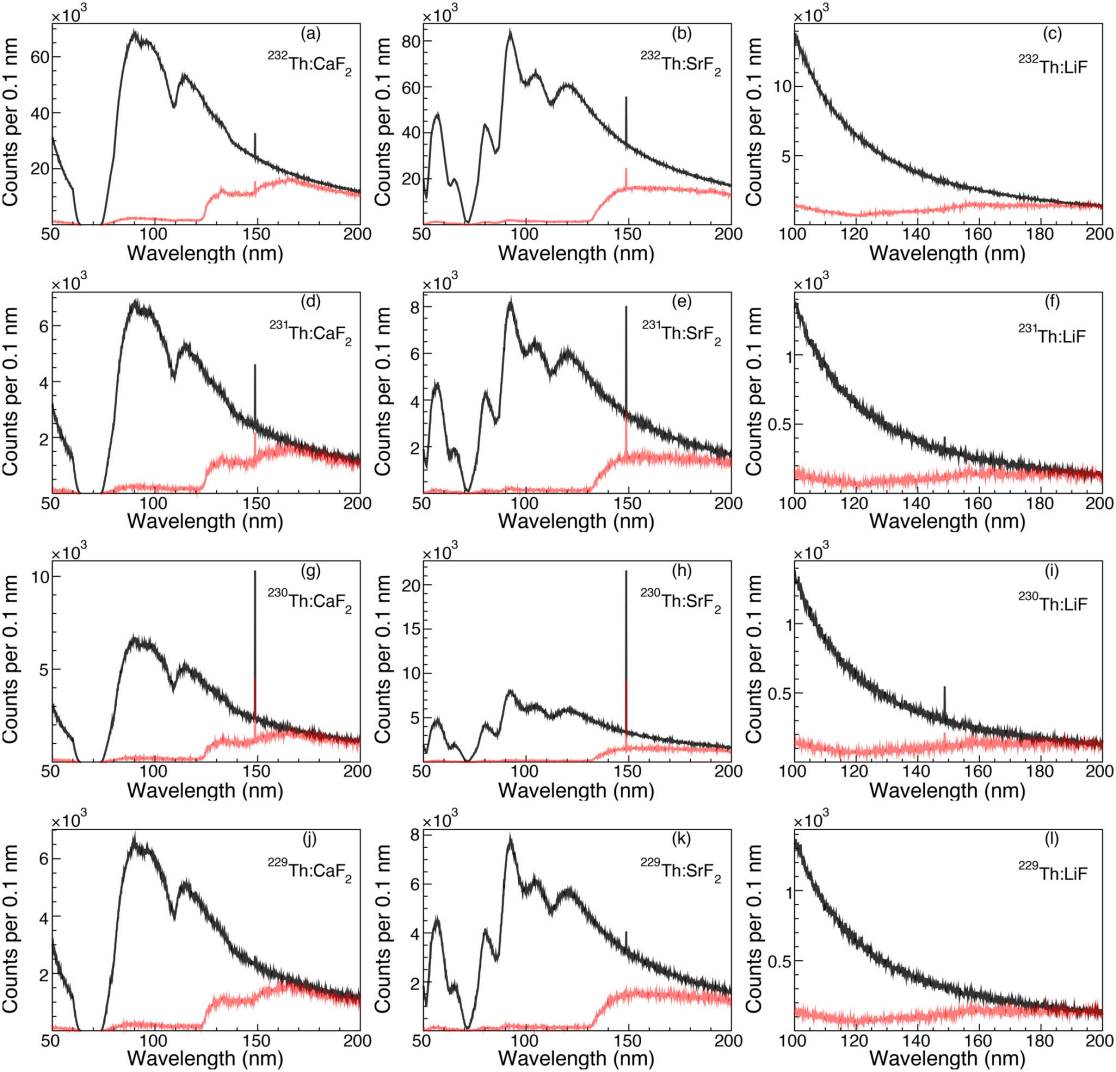
The spectra of optical photons generated inside (black lines) and emitted from (red lines) the thorium‐doped crystals in the time window of 1–10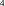 s after the irradiation of 1 nC electrons of 60 MeV. The latter is scaled by a factor of 2. (a)‐(l) are the photon spectra for 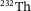:${\mathrm{CaF}}_{2}$, 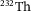:${\mathrm{SrF}}_{2}$, 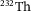:LiF, 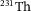:${\mathrm{CaF}}_{2}$, 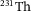:${\mathrm{SrF}}_{2}$, 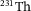:LiF, 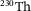:${\mathrm{CaF}}_{2}$, 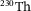:${\mathrm{SrF}}_{2}$, 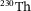:LiF, 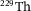:${\mathrm{CaF}}_{2}$, 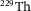:${\mathrm{SrF}}_{2}$, and 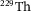:LiF, respectively. Here only the Cherenkov backgrounds from the photonuclear activation products are shown.

To provide a more intuitive presentation of the signal and backgrounds, 1‐D spectra of the optical photons generated inside and emitted from crystals doped with the four thorium isotopes at an electron energy of 60 MeV are shown in Figure 5. The signals for the ${\mathrm{SrF}}_{2}$ cases are more potent than those for the other crystals for the four doped isotopes. This is because the doping concentration of thorium in ${\mathrm{SrF}}_{2}$ is higher than that in ${\mathrm{CaF}}_{2}$ and LiF, and the Cherenkov background caused by the reaction residuals of Sr lies in a time window different from that of the 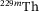 isomer. Moreover, the 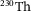 case exhibits better performance than all the other thorium isotopes, as its cross‐section for 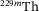 production is larger than the others.

### 2.3 Correlation of Signal‐to‐Noise Ratio and Incident Electron Parameters

To quantify the feasibility of performing VUV detection of 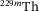 direct photon decay based on photonuclear excitation, the signal‐to‐noise ratios (SNRs) of the 1‐D wavelength spectra of the optical photons emitted from the crystals at different electron energies were extracted from the simulation results. The signal‐to‐noise ratio is defined as:

(3) $$SNR=\frac{S}{\sqrt{B}}$$

where S is the net counts of the 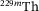 signal at 148 nm, and B is the background counts within the region of 148±5 nm, which is a conservative estimate that accommodates potential broadening mechanisms. The dominant contributions are the Doppler broadening of the 8.4 eV emission from 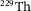 nuclei at room temperature ($∼10^{-4}$ nm) and the finite resolution of typical VUV spectrometers (generally < 1 nm).

**FIGURE 6:**
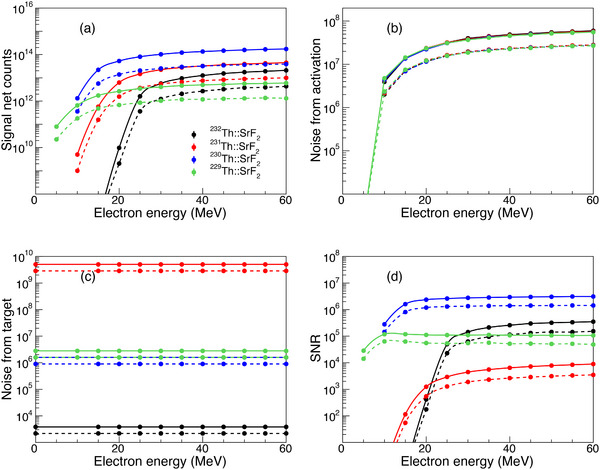
The correlation of the net signal counts (a), noise from photonuclear activation products (b), noise from the thorium dopants (c), and SNRs (d) against electron energy for the four thorium ${\mathrm{SrF}}_{2}$ crystals. Here, the solid lines and dashed lines stand for cases of the optical photons generated inside and emitted out of the crystals, respectively. The number of incident electrons is 1 C.

Figure 6a shows the dependence of the net signal counts on the energy of the incident electron with a charge of 1 C for the four thorium‐doped ${\mathrm{SrF}}_{2}$ crystals. Generally, the net signal counts grow with increasing electron energy and saturate around 40, 30, 25, and 20 MeV for the 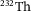, 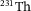, 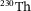, and 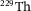 doped ${\mathrm{SrF}}_{2}$, respectively. The signal counts generated inside the target are nearly five times larger than those emitted out of the target because the optical photons are absorbed when traveling in the crystal. The maximum achievable signal counts (with the magnitude of 10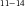) increase with the decreasing mass number of the thorium dopant, which is mainly caused by the 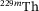 production cross‐sections. It is worth noting that 1 C of electron charge can be easily delivered by modern accelerator technology. Currently, electron intensities can reach the order of mA from conventional linear accelerators. For example, the ELBE at the Forschungszentrum Rossendorf, ARIEL at TRIUMF, and Linac‐200 at JINR Dubna routinely deliver electron beams with comparable or higher energies, and the intensity of ∼mA can be reached [55, 56, 57]. With an incident electron intensity of 1 mA, the required charge can be delivered within two half‐lives of the 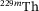 isomer. The corresponding 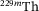 excitation rate can reach 1.28$\times 10^{10}$ $s^{-1}$, which is nearly three magnitudes higher than the NRS and VUV excitation(see Table 1). Moreover, laser‐wakefield electrons with energies of hundreds of MeV, charges of ∼nC/shot, and pulse widths of tens of fs can be produced by using high‐power lasers, which have been utilized for photonuclear cross‐section measurements and isomer production  [58, 59, 60, 61, 62]. Bremsstrahlung driven by such short‐pulse electrons can also be used to investigate intensity‐dependent 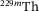 excitation properties such as the quenching effect  [32]. In such experiments, laser plasma‐induced electromagnetic pulses (EMP) in advanced accelerator systems constitute a transient noise source distinct from the persistent Cherenkov background. We note that such EMP noise typically lasts only tens of nanoseconds, whereas the detection of the 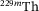 radiative decay is conducted over timescales of minutes to hours. By initiating measurements several seconds after activation, this transient noise can be effectively excluded.

**TABLE 1:** Characteristics and excitation rates of different sources. A typical photonuclear excitation rate of the :${\mathrm{SrF}}_{2}$ is used for comparison.

Excitation method	Source	Beam characteristics	Excitation rate ${[s}^{-1}]$
Direct excitation [33]	VUV laser	10 μJ, 30 Hz, Δ=2π×20 GHz	$2.1\times 10^{7}$
XRS [32]	Synchrotron	1.86 × 10 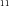 $s^{-1}$, Δ=30 meV	$4.5\times 10^{4}$
Photonuclear	Bremsstrahlung	1 mA, $E_{e}$ = 40 MeV	$1.28\times 10^{10}$

Figure 6b,c shows the correlation of electron energy and background noise originating from the radioactive decay of the photonuclear activation products and the thorium dopants, respectively. The noises from activation for the four ${\mathrm{SrF}}_{2}$ crystals are nearly the same and tend to rise with increasing electron energy. The maximum activation noise is of the order of $∼10^{8}$. The noises from the thorium dopants, on the other hand, depend mainly on the half‐life and β decay components, but not the incident electron energy. 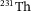:${\mathrm{SrF}}_{2}$ has the highest noise of ∼ 10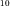 since 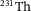 has a short half‐life of 25.52 h while 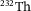:${\mathrm{SrF}}_{2}$ has the lowest noise of ∼ 10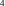 since 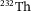 has a long half‐life of 1.4 × 10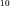 y. 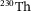:${\mathrm{SrF}}_{2}$ and 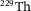:${\mathrm{SrF}}_{2}$ have a medium noise of $∼10^{6}$. As a result, the total noise of 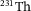:${\mathrm{SrF}}_{2}$ is predominantly from the radioactive decay of the thorium dopant.

Figure 6d shows the correlation of the electron energy and SNRs for the four thorium‐doped ${\mathrm{SrF}}_{2}$ crystals. The SNRs for 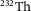:${\mathrm{SrF}}_{2}$, 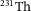:${\mathrm{SrF}}_{2}$, and 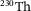:${\mathrm{SrF}}_{2}$ grows with electron energy and saturate around 40, 30, and 25 MeV, respectively. The SNR for 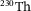:${\mathrm{SrF}}_{2}$ is the highest, reaching $∼10^{6}$ while that for 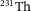:${\mathrm{SrF}}_{2}$ is of the order of $∼10^{3}$. Although the SNR for 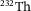:${\mathrm{SrF}}_{2}$ ($∼10^{5}$) is medium, it requires the least effort in target fabrication and is thus suitable for conducting preliminary experiments. In contrast, the SNR for 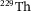:${\mathrm{SrF}}_{2}$ increases below 10 MeV and then decreases slightly as the electron energy grows. This behavior arises because the constant noise from the thorium dopants dominates below 10 MeV. As the energy increases, the noise from photonuclear activation takes over and grows simultaneously with the net signal counts, leading to the observed decrease and subsequent saturation of the signal‐to‐noise ratio. Note that the SNR values presented here are intrinsic figures of merit, calculated directly from the simulated emission spectra of the crystal itself, prior to interaction with any specific detection system. They serve as a universal benchmark for comparing different hosts and activation parameters when the exact experimental spectroscopy apparatus is unknown. The observed SNR in any experiment will be critically dependent on the efficiency of the specific detection apparatus. As detailed in Figure S4, weighting the intrinsic emission by realistic detection efficiency profiles  [32, 33] typically leads to an order‐of‐magnitude reduction in the observable SNR. In the context of VUV photon detection, Czerny‐Turner spectrometers can be employed to detect and identify 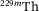 direct photon decay signals  [26, 33, 34]. VUV‐reflective parabolic mirrors can also be installed to enhance signal collection, as the signals are expected to be emitted isotropically in space. The background caused by the thorium dopants can be remedied by carefully subtracting the pre‐measured target background spectra from the post‐irradiation spectra, or by anti‐coincidence of backgrounds and signals [32].

It is important to note that the β/γ radiation emitted by radioactive products can affect crystal performance or pose handling concerns. Available experimental evidence suggests that the degradation of VUV transmission due to such radiation is manageable. For instance, an experiment was performed in Ref. [37], which irradiated a ${\mathrm{CaF}}_{2}$ crystal with reactor neutrons of 10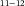 n s^−1^ for 3 h. Such intense neutron irradiation produces significant radioactive byproducts that emit β/γ radiation, and the associated neutron‐capture cross‐sections are typically orders of magnitude larger than those for photonuclear reactions. After irradiation, the crystal developed an orange coloration, but the VUV transmission did not decrease drastically—at most by a factor of 0.2. Refs. [37] and [38] further indicate that radioactivity can amplify fluoride loss during crystal growth, reducing VUV transparency and limiting the achievable 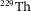 concentration. Nevertheless, VUV transmittance can be partially restored by proper high‐temperature annealing. As mitigation strategies, degradation of VUV transmission caused by radioactive decay radiation can be remedied by superionic fluoride transfer, i.e., annealing the crystals at 1250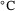 in ${\mathrm{CF}}_{4}$ gas, as demonstrated in Ref. [39]. Moreover, because most radioactive byproducts are short‐lived, the radiation dose can be substantially reduced by allowing the activated crystal to cool for a suitable period after irradiation, as it was partially verified by a preliminary experiment and its benchmarking simulations (see the Supporting Information).

In our simulations, we have incorporated experimentally measured VUV transmittance and absorption‐length data from the literature  [63, 64, 65, 66, 67, 68] to model photon transport. This approach is standard when first‐principles calculations of defect‐related absorption are not feasible for complex doped materials. We fully acknowledge that crystal defects—which depend on specific growth conditions and post‐processing—can significantly influence VUV transparency. A comprehensive, first‐principles modeling of defect‐induced absorption is a substantial research topic in itself and lies beyond the scope of the present study. Therefore, the absorption data used here represent values measured under reported growth conditions, and we note that defect concentrations—and hence VUV performance—may vary and require optimization in future experiments.

### 2.4 Yields in the Crystals

Photonuclear reactions offer a dual utility: beyond direct population and VUV detection of 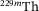, they enable in situ conversion of 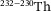
‐doped crystals into 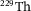
‐enriched variants. This addresses the critical supply chain vulnerability posed by the disposal of 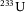 stockpiles—the historical source of 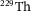. Three primary isotopic pathways emerge for crystal doping. First, 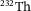, constituting 99.98% of natural thorium, provides an abundant and readily accessible feedstock for photonuclear conversion in crystals like ${\mathrm{CaF}}_{2}$ and ${\mathrm{SrF}}_{2}$. Second, 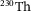, obtainable via chemical extraction from 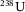 decay chains (natural abundance >99%), presents a scalable alternative if purification techniques advance. Finally, 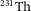, which requires artificial production via nuclear reactions, remains impractical for large‐scale applications.

**FIGURE 7:**
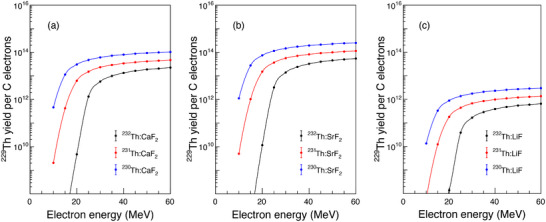
The 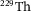 yield as a function of electron energy for the 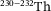
‐doped crystals of ${\mathrm{CaF}}_{2}$ (a), ${\mathrm{SrF}}_{2}$ (b), and LiF (c). The number of incident electrons is 1 C.

To quantify the production efficiency within candidate host materials, we have simulated the 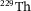 yields specifically within 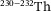
‐doped ${\mathrm{CaF}}_{2}$, ${\mathrm{SrF}}_{2}$, and LiF crystals (see Figure 7). The yield's dependence on incident electron energy follows a characteristic increase‐then‐saturate trend. Crucially, the total 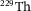 yield (the sum of ground state 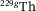 and isomer 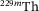) is typically a factor of 1–2 higher than the yield of the isomer alone. Two key material trends emerge from simulations: the saturated yield increases with decreasing mass of the target thorium isotope (i.e., 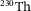 > 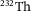), and it scales linearly with the thorium doping concentration in the crystal.

For a 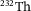:${\mathrm{SrF}}_{2}$ system, simulations indicate that an incident electron charge of 1000 C generates 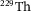 concentrations of $∼10^{15}$ ${\mathrm{cm}}^{-3}$ — sufficient for nuclear clock operation. The core quantitative relationship governing production is that the yield of 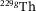 scales linearly with the incident bremsstrahlung photon flux, and thus with the total electron charge delivered to the converter target. This well‐established photonuclear relationship (see Equation (4)) forms the basis of our simulations. The extended 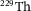 half‐life (7907 years) relaxes accelerator requirements, allowing dose delivery over extended periods via low‐average‐current beams (e.g., nA level). The electron beam parameters (e.g., 40 MeV energy) were selected to efficiently generate photons above the reaction threshold while remaining compatible with compact accelerator infrastructure, enhancing practical feasibility. The total charge represents a scalable target; the instantaneous beam current and irradiation time can be adjusted to manage thermal load and stay below the sample's damage threshold.

It is important to distinguish between the stability of the produced 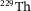 nuclei and potential radiation‐induced changes to the crystal's optical properties. The 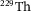 nuclei produced via the photonuclear reaction are embedded within the crystal and are not subject to loss from the crystal bulk, aside from their intrinsic radioactive decay. Note that the lower 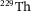 doping concentration compared to 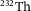 in ${\mathrm{CaF}}_{2}$ host, as observed in the crystal growth experiments Ref. [37, 38], is a deliberate choice made to balance the need for a high 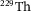 density against the requirement of maintaining sufficient VUV transmittance in the crystal. This compromise is not due to a loss of 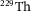 from fluoride depletion. Even after annealing in a ${\mathrm{CF}}_{4}$ atmosphere, the 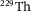 concentration in a crystal remained unchanged [39, 69, 70]. Consequently, any reduction in VUV transmittance due to effects like fluorine vacancy formation is a separate materials issue, not an indicator of 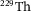 loss. To mitigate such radiation damage, host crystals like 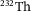:${\mathrm{CaF}}_{2}$ can be subjected to a ${\mathrm{CF}}_{4}$ annealing process during growth, which significantly improves radiation tolerance by pre‐emptively reducing fluorine vacancy concentration  [67]. Hosts like ${\mathrm{SrF}}_{2}$ are also under investigation for their inherently more radiation‐hard lattice structures  [66].

This method provides a potential pathway to create 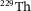
‐doped crystals from more readily fabricated 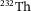
‐doped precursors. Direct experimental validation of the production yield is crucial and can be achieved through complementary methods. Offline activity measurements of the long‐lived 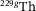 or short‐lived 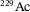, using techniques such as high‐purity germanium (HPGe) gamma spectroscopy, offer a definitive and quantitative method to confirm successful activation and to precisely determine production yields. We also emphasize that the comprehensive evaluation of ultimate solid‐state nuclear clock performance metrics, such as crystal microstructure, defect formation, frequency stability, and systematic error, constitutes a paramount long‐term objective for the field  [48, 71, 72, 73, 74]. Achieving these metrics requires overcoming significant experimental challenges, including direct VUV laser/comb excitation and spectroscopy of the 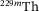 isomer within the crystal host—a capability currently available in only a few advanced laboratories. The present work establishes a foundational material production pathway. The subsequent characterization of the clock performance for crystals produced via this method is a critical and logical next phase of research, building directly upon the foundation laid here.

## 3 Conclusion and Discussion

This theoretical study proposes and evaluates a novel methodology for populating 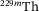 and detecting its VUV decay through photonuclear reactions within thorium‐doped crystals. We have established a complete physics model and simulation framework, progressing through the essential “theoretical calculation” and “simulation prediction” stages of research. TALYS calculations were performed to evaluate the cross‐sections for photonuclear reactions leading to 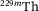
‐related residuals, with results indicating that population cross‐sections can reach the order of hundreds of millibarns. Subsequent Geant4 simulations were conducted to estimate the prospect of detecting the direct photon decays of 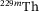 from 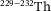
‐doped ${\mathrm{CaF}}_{2}$, ${\mathrm{SrF}}_{2}$, and LiF crystals. The time‐wavelength distributions of the optical photon signals and Cherenkov radiation backgrounds were simulated, and the correlation between the signal‐to‐noise ratio and incident electron energy was analyzed. Our simulations predict that a high excitation rate on the order of $∼10^{10}$ $s^{-1}$ could be achieved using a moderate electron beam current of 1 mA. Furthermore, the simulations indicate that thorium‐doped ${\mathrm{SrF}}_{2}$ offers superior performance among the candidates, yielding a theoretical SNR on the order of $10^{6}$ under an irradiation of 1 C of ∼40 MeV electrons. This proposed method offers distinct theoretical advantages, including unique target material flexibility, compatibility with broad‐spectrum bremsstrahlung sources, and predicted excitation rates significantly higher than those of VUV laser direct excitation or X‐ray pumping methods. Crucially, this approach presents a sustainable pathway by enabling the in situ conversion of abundant 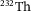 and 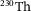 into 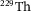
‐doped crystals, thereby potentially bypassing future reliance on scarce isotopes and addressing material supply challenges.

Note that the primary contribution of this work is the establishment of a detailed theoretical groundwork that defines a new approach, establishes its principle feasibility through simulation, and provides a clear roadmap for subsequent experimental validation. We believe this foundation offers significant and timely value to the field. The logical next phase of research, which builds upon this theoretical framework, involves the comprehensive experimental validation of the production yields, a detailed microstructural characterization of irradiated crystals, and a rigorous benchmarking of the simulation models against precise dosimetry and activity measurements. This future work will be essential to close the scientific loop and fully assess the practical potential of photonuclear reactions for creating and characterizing 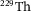
‐doped crystalline materials for fundamental physics and precision metrology.

## 4 Methods

### 4.1 Photonuclear Cross‐Sections Calculations

To evaluate the 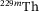 production cross‐section, theoretical calculations were performed on the photonuclear reactions on isotopic targets of 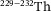 using Talys, which provided a complete description of all reaction channels and observables, and many state‐of‐the‐art nuclear models covering all the main reaction mechanisms encountered in light‐particle‐induced nuclear reactions were included. The nuclear structure components used in the TALYS calculations were as follows: The nuclear masses were taken from the Atomic Mass Evaluation 2020 (AME2020) [75] whenever available, whereas the latest version of the Skyrme‐Hartree‐Fock‐Bogoliubov nuclear mass model by Goriely and Pearson  [76] were taken into account when the AME2020 mass data was not available. The discrete experimental levels compiled in the RIPL‐3 library [77] and the continuum level spectrum represented by the nuclear level densities (NLDs) were both considered in the calculations. The NLDs were obtained from predictions in the Fermi gas model plus a constant temperature  [78]. The photon strength functions (SFs) obtained from the phenomenological Simplified Modified Lorentzian Model (SMLO) were used to calculate the electromagnetic transmission coefficients for the photon channel. The phenomenological optical model potentials (OMPs), i.e., the local and global parameterizations of Koning and Delaroche were employed to determine the transmission coefficient for the particle channels  [79]. Specifically, the half‐life of the 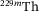 isomer was set as 7.6 μs and the internal conversion coefficient was set as 10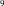. To verify the reliability of these nuclear models, the calculated cross‐sections of thorium isotopes were compared with the available experimental data in the EXFOR database, see Figure S1. Also, the uncertainties of the calculated isotope and isomer production cross‐sections were evaluated with the TASMAN code (see Figure S2), which was a computer code system for nuclear data uncertainty distributions based on the nuclear model code TALYS.

### 4.2 Reaction Yield of Related Residuals

When isotopically pure 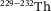 targets were irradiated by bremsstrahlung radiations, the 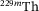
‐related radioactive product yields can be estimated by

(4) $$Y=\frac{\rho N_{A}}{M}\int \sigma \left(E_{\gamma }\right)\Phi \left(E_{\gamma },E_{e}\right)\frac{1-exp[-\mu \left(E_{\gamma }\right)L]}{\mu (E_{\gamma })}dE_{\gamma }$$

where $E_{e}$ is the incident electron energy, $E_{\gamma }$ is the bremsstrahlung photon energy, ρ is the target density, $N_{A}$ is the Avogadro constant, M is the atomic mass of the target, L is the target thickness, $\sigma (E_{\gamma })$ is the calculated photonuclear cross‐section, $\Phi (E_{\gamma },E_{e})$ is the bremsstrahlung energy distribution, $\mu (E_{\gamma })$ is the linear attenuation coefficient of photon propagating in the Thorium target. Simulations were also performed to evaluate the yield of 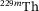 and its related radioactive residuals under bremsstrahlung irradiation. The calculated photonuclear reaction cross‐sections were incorporated into the Geant4‐GENBOD toolkit to model the photonuclear process. The G4LowEGammaNuclearModel models the radioactive fragmentation from (γ, f) reactions and the photonuclear reactions on other materials. The simulation configuration was arranged as follows: A pencil electron beam hits a Ta convertor with a thickness of 2 mm, and the resulting bremsstrahlung irradiates the photonuclear targets, which were composed of isotopically pure 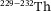 with a cylindrical shape of ϕ2 × 1 cm and a density of 10 g ${\mathrm{cm}}^{-3}$. The electron beam energy was scaned in the range of 5–150 MeV in steps of 2 MeV.

### 4.3 Optical Effects and Cherenkov Backgrounds in Crystal

In order to investigate the feasibility of performing VUV detection of 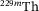 direct photon decay based on photonuclear excitation of thorium isotopes embedded inside such crystalline environments, Geant4 simulations were also performed in this work. In the simulations, the basic configurations used in Section 4.2 were kept unchanged, and a few modifications were given according to the current investigation requirements. The electron beam impinges a Ta converter, inducing bremsstrahlung radiation. The electron beam energy was scaned in the range of 5–60 MeV in steps of 5 MeV. A activation target (crystal) was placed right behind the converter, which was a crystal consist of ${\mathrm{SrF}}_{2}$  [66], ${\mathrm{CaF}}_{2}[$ 67], or LiF [68] doped with isotopically pure 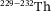. These crystals were recently developed for 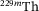 experiments, which comprise a high doping concentration of 10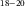 ${\mathrm{cm}}^{-3}$ and an acceptable photon transmittance of 50–70 % around the wavelength of 148 nm. These crystals' dimensions were set to cylinders of ϕ1 × 1 cm. The doping concentration of 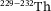 isotopes in Th:${\mathrm{CaF}}_{2}$, Th:LiF, and Th:${\mathrm{SrF}}_{2}$ crystals used in the simulations are listed in Table 2. Note that comparable doping levels had been achieved in other key studies. For instance, Beeks et al. [37, 38, 39] successfully grew 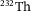:${\mathrm{CaF}}_{2}$ crystals with concentrations in the range of 4.0$\times 10^{19}$–2.6$\times 10^{20}$ ${\mathrm{cm}}^{-3}$, which were consistent with the concentrations used in our work. Their 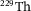:${\mathrm{CaF}}_{2}$ crystals had been widely employed in recent groundbreaking experiments toward solid‐state nuclear clock development. It was worth noting that in practice, 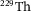 concentrations tend to degrade to 10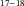 ${\mathrm{cm}}^{-3}$ due to radioactivity amplified fluoride loss in ${\mathrm{CaF}}_{2}$ during growth [37, 38, 39]. A similar effect would be expected in our studied 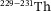
‐doped systems. While our current doping concentration settings represent ideal estimates for theoretical modeling, we emphasized that actual experimental concentrations may vary for such 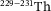
‐doped crystals, and such deviations should be carefully considered in practice.

**TABLE 2:** The doping concentration of isotopes in Th:${\mathrm{CaF}}_{2}$, Th:LiF, and Th:${\mathrm{SrF}}_{2}$ crystals.

Dopant isotope	Hosting crystal	Concentration (atoms/${\mathrm{cm}}^{3}$)
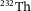	${\mathrm{CaF}}_{2}$	2.76 × 10 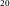
	LiF	8 × 10 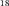
	${\mathrm{SrF}}_{2}$	6.7 × 10 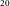
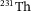	${\mathrm{CaF}}_{2}$	2.76 × 10 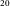
	LiF	8 × 10 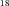
	${\mathrm{SrF}}_{2}$	6.7 × 10 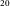
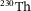	${\mathrm{CaF}}_{2}$	2.76 × 10 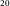
	LiF	8 × 10 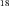
	${\mathrm{SrF}}_{2}$	6.7 × 10 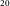
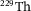	${\mathrm{CaF}}_{2}$	2.76 × 10 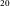
	LiF	8 × 10 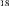
	${\mathrm{SrF}}_{2}$	6.7 × 10 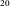

The resulting 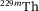 direct photon decay signal and the Cherenkov backgrounds were diagnosed. To model the radioactive decay of the photonuclear activation products, the G4RadioactiveDecay class was implemented. A G4GammaToOptical class was constructed and registered to convert the γ decay signals of 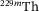 into optical photons since its decay photon lied in the optical region. To transport the optical photons and estimate the backgrounds caused by optical effects, G4OpticalPhysics was used in the physics module. The refractive indices data from Refs.  [63, 64, 65] are used for the Cherenkov effects caused by charged particles and the transmittance data from Refs.  [66, 67, 68] were employed for the absorption of optical photons inside the crystals (see Figure S3). Note that the physics of Cherenkov radiation generation was fundamental and implemented in Geant4 (G4Cerenkov class in the G4OpticalPhysics module) according to the Frank‐Tamm formula, which was reliably used in diverse fields from particle physics to medical imaging. Our simulation chain builds upon these validated components. The key input to this chain—the production cross‐sections of the radioactive isotopes—had been benchmarked against experimental nuclear data and sensitivity analysis of input nuclear parameters were performed, as shown in Figures S1 and S2. We also note that the estimation of Cherenkov radiation in thorium‐doped crystals had been studied using conceptually similar approaches. For example, seminal works by Beeks et al. [37] and Kraemer et al. [23, 24] had incorporated key parameters—such as beta‐decay energy spectra from theoretical calculations, energy loss of charged particles from SRIM simulations and crystal refractive indices from the literature—into the Frank‐Tamm formula for their calculations. Our full‐scale simulation, which self‐consistently tracks the process from radioactive decay to Cherenkov photon emission, provided a more comprehensive and integrated description of the underlying physics.

## Conflicts of Interest

The authors declare no conflicts of interest.

## Supporting information


**Supporting File**: advs74600‐sup‐0001‐SuppMat.pdf.

## Data Availability

The data that support the findings of this study are available from the corresponding author upon reasonable request.
