# Supplementary material for: Photonuclear Population of 229m,gTh in Th‐Doped Crystals Toward Nuclear Clock Development
Source: Adv Sci (Weinh). 2026 Feb 27;13(26):e23384. doi: 10.1002/advs.202523384 (PMC13159134; doi:10.1002/advs.202523384)
Supplement: Supplementary file 1 — Supporting File: advs74600‐sup‐0001‐SuppMat.pdf. [file ADVS-13-e23384-s001.pdf]

Supplementary Material

Verification of nuclear models and uncertainty of the Talys calculations

To check the reliability of the nuclear models used in our Talys calculations, we benchmark our theoretical cross-section calculations against available experimental data from the EXFOR database, as well as the evaluated data from IAEA PD-2019, JENDL 5.0, TENDL-2023, and ENDF-VIII. Current experimental photonuclear data for thorium isotopes are mainly limited to the (γ, n), (γ, 2n), and (γ, 3n) channels on ^{232}Th. As shown in Fig. S1, our TALYS calculations show fair agreement with these datasets and successfully reproduce the Giant Dipole Resonance (GDR) structure of ^{232}Th. Notably, the (γ, 3n) cross section—directly related to ^{229m,g}Th production—aligns better with experimental values than the evaluated data, supporting the reliability of our theoretical framework for the key reaction channel.

Moreover, the uncertainties of the calculated cross sections for the production of ^{229m,g}Th, ^{229}Ac, and ^{229}Ra are further evaluated. We incorporate the propagated effects of uncertainties in fundamental inputs—such as the optical model potential (OMP), nuclear level densities (NLD), and gamma-ray strength functions (SF). To quantify this, a systematic uncertainty propagation—for example, using the TASMAN code—varies these parameters within their established covariance ranges. While the main text presents the central-value results, we have performed this analysis. The associated uncertainty band (see Fig. S2), typically within one order of magnitude in the GDR region for photonuclear reactions, reflects the collective variation of all model parameters. Note that using broadband bremsstrahlung to induce photonuclear reactions, the reaction yield is a convolution of cross section and the photon spectrum that mainly affected by the cross sections in the peak regions.

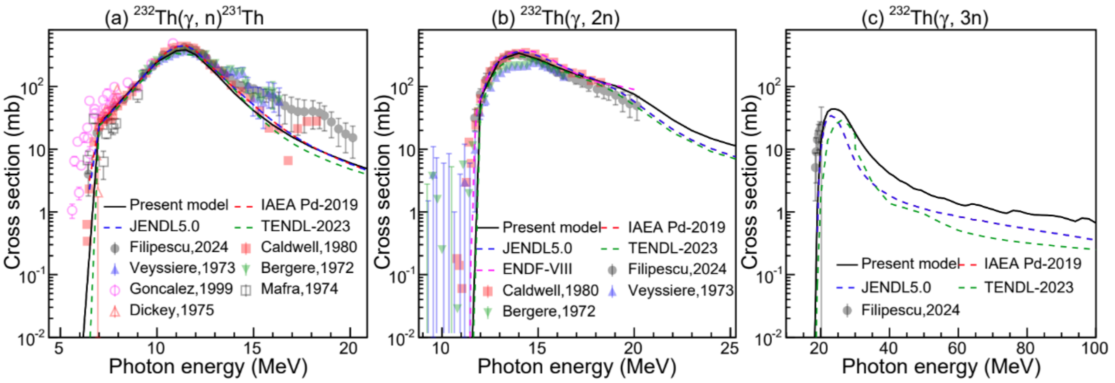

Fig. S1 (a), (b), and (c) compare the photonuclear reaction cross sections calculated by Talys with experimental data from the EXFOR database for the ^{232}Th(γ,n), ^{232}Th(γ,2n), and ^{232}Th(γ,3n) reactions, respectively. The solid black line shows the theoretical Talys results, while the data points with error bars represent experimental EXFOR data from various sources.

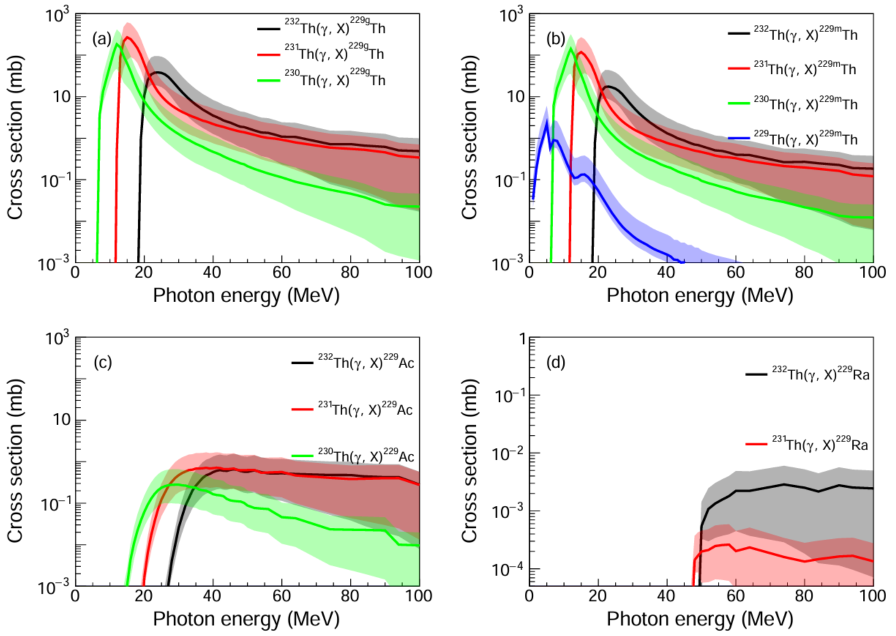

Fig. S2 The uncertainty of photonuclear cross section to produce ^{229g}Th (a), ^{229m}Th (b), ^{229}Ac (c), and ^{229}Ra (d), resulting from the uncertainty of the input nuclear parameters of NLD, SF, and OMP. The uncertainty calculation is estimated using TASMAN software.

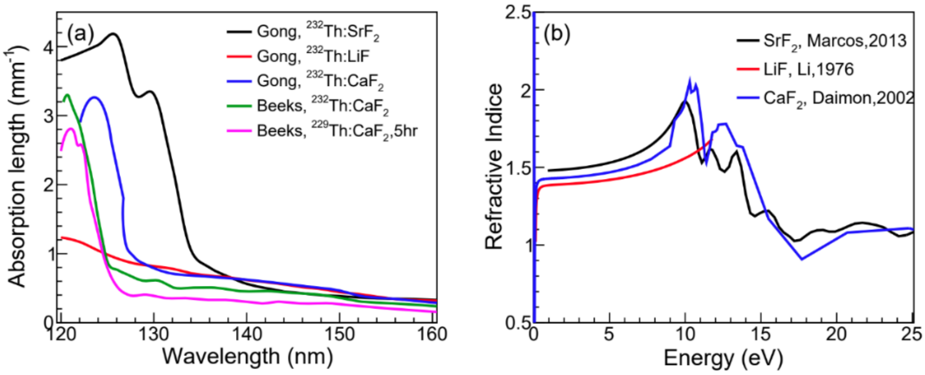

Fig. S3 (a) The experimental absorption lengths for the ^{232}Th:SrF_2 , ^{232}Th:LiF and ^{232}Th:CaF_2 crystals from the results of Gong *et al.* [Refs. 65-67] used in the simulations of absorption of optical photons, along with the those for ^{232}Th:CaF_2 and ^{229}Th:CaF_2 after annealing with CF_4 gas from the results of Beeks *et al*. [Ref. 39]. (b) The experimental refractive indexes for the SrF_2 , LiF and CaF_2 crystals used in the simulations of generation of optical Cherenkov photons.

Refractive index and transmittance used in the optical simulations

The Cherenkov background originates from charged particles generated by radioactive decays following photonuclear reactions as they travel through the crystal—a process intrinsically linked to the material's refractive index. In our simulations, the production of radioactive isotopes is modeled using our calculated reaction cross-sections with validated nuclear models, while the resulting charged particles from their decays are handled by a realistic radioactive decay physics module. The ensuing Cherenkov radiation is simulated based on existing experimental refractive index data (Fig. S3 (b)). We note that such similar approach—combining refractive index data with the Frank-

Tamm formula—has been extensively used in prior foundational studies, such as those by Beeks *et al.* [Refs. 37] and Kraemer *et al.* [Refs. 23, 24], to estimate Cherenkov backgrounds in ^{229}Th-doped or ^{229}Ac-implanted CaF_2 crystals. Furthermore, to accurately model photon transport and absorption within the crystals, we incorporated experimental absorption lengths (Fig. S3 (a)) into the optical photon propagation routines.

Expected SNR under realistic detection systems

The SNR value presented in our manuscript represents the "*intrinsic*" SNR of the photon signal generated and emitted from the crystal itself, prior to interaction with any specific detection system. This intrinsic metric is calculated directly from our simulated emission spectra and is intended as a fundamental, system-agnostic figure of merit for comparing different crystal hosts and activation parameters.

The "*observed*" SNR in any real experiment is critically dependent on the specific detection apparatus. To bridge this gap and address this issue, we have performed a detailed analysis, as summarized in Fig. S4. The figure shows the detection efficiency profiles for two representative systems: one typical of X-ray pumping experiments (centered at 150 nm) and another for direct VUV laser excitation (centered at 146 nm). Weighting our simulated intrinsic emission spectra by these efficiency curves yields the spectra that would actually be recorded.

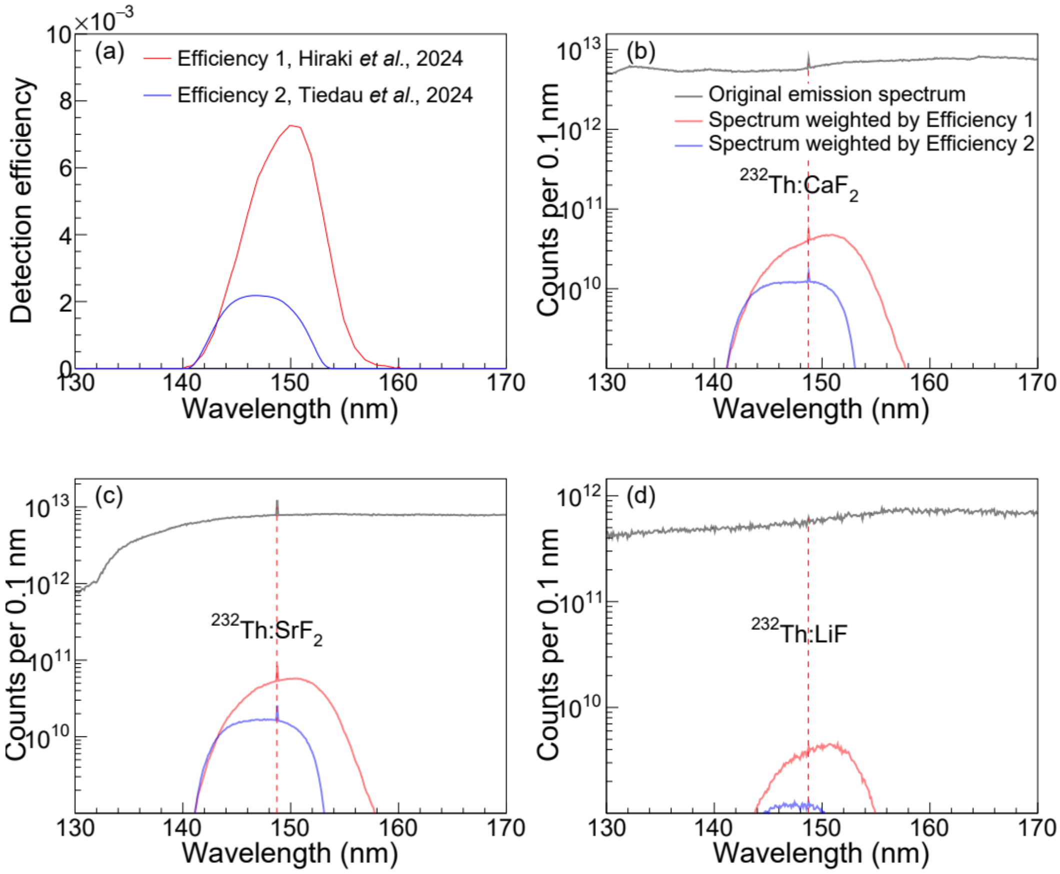

Fig. S4 (a) Overall detection efficiency for typical systems used to detect the decay of ^{229m}Th in X-ray pumping [Ref. 32] (red solid line) and direct VUV laser excitation [Ref. 33] (blue solid line) experiments.

(b–d) Comparison of the original optical photon emission spectra from $^{232}Th:CaF_2$ (b), $^{232}Th:SrF_2$ (c), and $^{232}Th:LiF$ (d) at incident electron energy of 60 MeV and charge of 1 C with the corresponding spectra weighted by the two detection efficiencies shown in (a).

The impact is significant and quantifiable:

[1] For $^{232}Th:CaF_2$, the intrinsic SNR in the 148±5 nm window is 7.6×10^4. When convolved with the X-ray pumping detection profile (140-160 nm window), it decreases to 6.6×10^3. With the VUV laser excitation profile (140-155 nm window), it further decreases to 3.3×10^3.

[2] For $^{2232}Th:SrF_2$, the intrinsic SNR of 1.6×10^5 decreases to 1.4×10^4 and 7.9×10^3 for the respective detection systems.

[3] For $^{232}Th:LiF$, the signal in this region is negligible in both the intrinsic and efficiency-weighted spectra.

These results demonstrate that incorporating realistic detection efficiencies typically leads to an order-of-magnitude reduction in the observable SNR, with the exact value depending on the specific detector and crystal host.

Therefore, providing the intrinsic SNR serves a valuable purpose: it establishes a universal, comparable benchmark. Individual experimental groups can then conveniently combine this benchmark with their own system's characterized efficiency (including effects like spectral overlap and error) to obtain a practical estimate tailored to their setup.

Preliminary experiment results

As suggested, we present results of a preliminary gamma irradiation experiment using the $^{232}Th:CaF_2$ crystal (1 cm diameter × 2 mm thickness). Under irradiation of a 500 pA, 40 MeV electron beam for 20 min, the $^{232}Th:CaF_2$ crystal also takes on an orange color, and the transmission before and after the irradiation are measured. Moreover, the radiation dose right after the irradiation was in the order of μSv/h and it falls back to the order of natural background dose (~0.11 μSv/h) within one day, because the majority of the short-lived radioactive products have decayed. Note that the preliminary experiment reported here was conducted primarily to demonstrate the basic feasibility of the proposed activation route, providing practical justification for this theoretical exploration.

A comparison of the VUV absorption length—which directly reflects transmittance—for the same crystal under these two conditions, alongside reference data for $^{232}Th:CaF_2$ and annealed $^{229}Th:CaF_2$ from Ref. [39], as well as for $^{232}Th:SrF_2$ Ref. [67] and $^{232}Th:LiF$ Ref. [68] (see Fig. S5). The wavelength region of 140-160 nm was selected because it fully covers the expected ^{229m}Th decay signal near 148 nm. A 20 nm window is sufficiently wide to accommodate potential shifts or splitting of the isomer energy level, as well as the accompanying Cherenkov background. These results can therefore provide practical guidance for future ^{229m}Th radiative-decay measurements and direct

VUV laser excitation experiments. The observed degradation in VUV transmittance after irradiation is likely attributable to fluorine migration within the crystal lattice under radiation exposure, leading to the formation of Ca metallic colloids and thorium dopants in altered surroundings and charge states. As reported in Refs. [38, 39] and supported by prior studies Refs. [69, 70], Th ions in different local environments produce absorption around 130 nm, while Ca colloids contribute to broad absorption near 150 nm. The superposition of these effects results in an overall increase in absorption across the 140-160 nm range. Additionally, in our preliminary experiment, the crystal developed an orange coloration—similar to that observed in neutron-irradiated CaF_2 and in long-term colored $^{229}Th:CaF_2$. This coloration is primarily caused by the formation of F, M, and N centers, which absorb around 400 nm and emit near 600 nm, giving rise to the orange appearance.

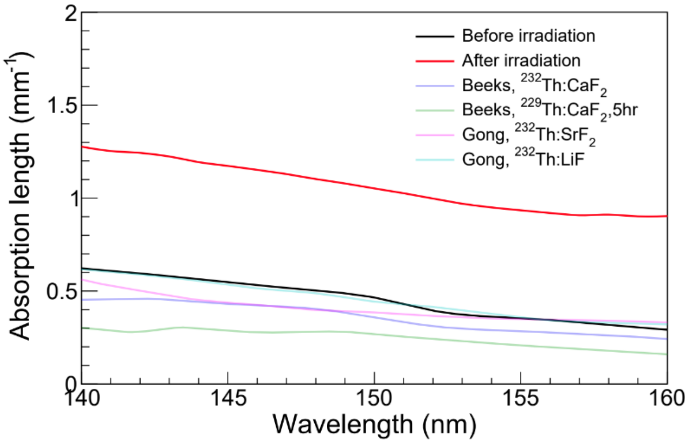

Fig. S5 The absorption length of the $^{232}Th:CaF_2$ crystal in the region of 140-160 nm before (black line) and after (red line) the electron beam irradiation. The results for $^{232}Th:CaF_2$ and annealed $^{229}Th:CaF_2$ from Beeks *et al*. Ref. [39] are also shown for comparison, as well as those of the $^{232}Th:SrF_2$ Ref. [67], and $^{232}Th:LiF$ Ref. [68] crystals.

Regarding the quantitative distribution of radiation dose over time, we have performed verification simulations based on the exact electron-beam and crystal parameters used in our preliminary experiment. The results are presented in Fig. S5. First, the simulated ambient dose equivalent rate for the $^{232}Th:CaF_2$ crystal is ~μSv/h immediately after irradiation and decays to ~0.1 μSv/h after one day (8640 s) of cooling (Fig. S6 (a)). This trend is consistent with the dose-rate variation observed experimentally. For comparison, we also simulated the ambient dose rates for $^{232}Th:SrF_2$ and $^{232}Th:LiF$ crystals under identical irradiation conditions. All three crystals show a similar pattern: the dose rate decreases gradually up to about 10^4 s, then decays nearly exponentially to ~10^{-3} μSv/h at 10^6 s. The dose rate for $^{232}Th:SrF_2$ is slightly higher than that for $^{232}Th:CaF_2$, while that for $^{232}Th:LiF$ is slightly lower; all remain within the same order of magnitude. These results indicate that a cooling period of several days is sufficient to reduce the dose rate to a level that permits safe handling for subsequent crystal property measurements. In addition, although a full theoretical explanation of the crystal coloration and VUV transmission changes would require a very dedicated study, we provide the time evolution of the accumulated radiation dose absorbed by the

crystals as auxiliary data for future investigations (Fig. S6(b)). The absorbed dose increases slowly up to about 10^{11} s, then rises sharply and saturates beyond 10^{14} s. This behavior directly reflects the composition of the radioactive inventory shown in Fig. 4(a-d): the initial contribution comes from short-lived γ/β emitters, whereas the dominant long-term dose is due to long-lived α emitters such as ^{229}Th and ^{230}Th (half-lives ~10^{11} s). Consequently, the long-term radiation environment in the irradiated crystal resembles that of a crystal intentionally doped with purely ^{229}Th. Furthermore, these results also provide a direct verification analysis of simulation results based on experimental data of the radiation dose, as the referee also concerns.

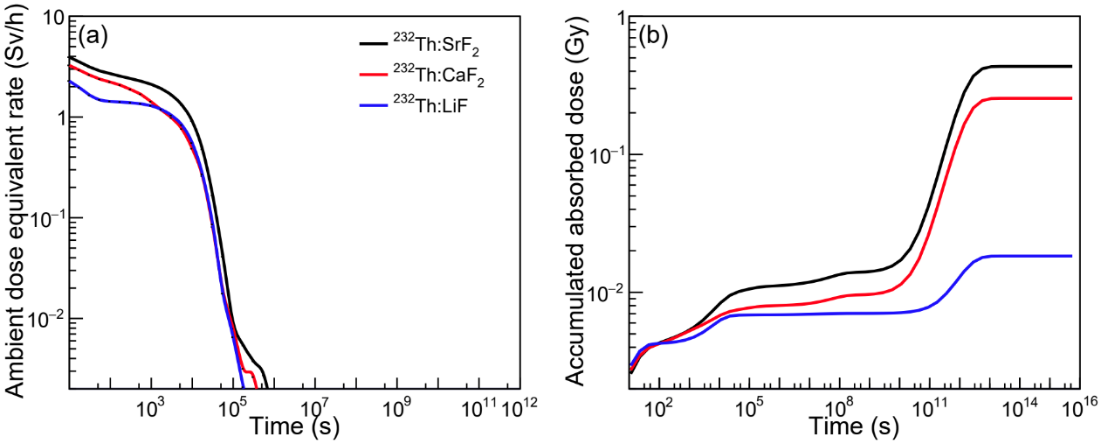

Fig. S6 Time evolution after bremsstrahlung irradiation of (a) the ambient dose equivalent rate at a distance of 2 cm from the crystal and (b) the accumulated radiation dose absorbed by the crystal. Simulations were performed for an electron energy of 40 MeV and a total charge of 0.6 μC, corresponding to a 500 pA beam delivered over 20 min. The crystal dimensions (1 cm diameter × 1.5 mm thickness) match those of the sample used in the preliminary experiment.

We acknowledge that quantifying the continuous, long-term impact of embedded radioactivity on VUV transmittance and structural integrity is a complex materials science question. While our present study does not provide a complete theoretical model for this, the calculated time evolution of the absorbed dose (Fig. S6(b)) serves as a crucial quantitative input for future dedicated studies on radiation damage dynamics. Regarding the proposed CF_4 annealing repair strategy, our analysis offers strong supportive evidence. The long-term absorbed dose is dominated by alpha-emitting isotopes like ^{229}Th and ^{230}Th. Critically, the existing literature provides direct experimental support for the efficacy of CF_4 annealing in repairing the specific damage caused by thorium and its decay chain. The works of Ref. [39,66] have demonstrated that the VUV transmission degradation in ^{d}Th-doped fluoride crystals—resulting from mechanisms such as fluorine vacancy formation—is largely recoverable via CF_4 annealing. This establishes a clear precedent for the practicality of the approach we discuss.

We also note that characterizing crystal microstructure stability is a critical aspect of developing viable nuclear clock crystals. We acknowledge, however, that this presents a profoundly complex challenge, as it requires disentangling the interrelated effects

such as crystal defects, temperature, radiation exposure, and long-term VUV irradiation on material properties, which would require more complex characterizations rather than just only XRD or Raman spectroscopy. Currently, only a very limited number of research groups worldwide possess the integrated capabilities to conduct such systematic studies, with recent efforts primarily focused on $^{229}Th:CaF_2$, as reflected in recent works such as Refs. [48, 71, 72, 73, 74]. Given the variety of crystal hosts and dopants explored in our broader study, obtaining definitive and universally applicable conclusions on microstructural stability extends beyond the scope of a single paper—or even the work of one research team. It necessitates sustained, collaborative effort across the scientific community.
